# Pyruvate Dehydrogenase Kinase 4 Promotes Vascular Calcification via SMAD1/5/8 Phosphorylation

**DOI:** 10.1038/srep16577

**Published:** 2015-11-12

**Authors:** Sun Joo Lee, Ji Yun Jeong, Chang Joo Oh, Sungmi Park, Joon-Young Kim, Han-Jong Kim, Nam Doo Kim, Young-Keun Choi, Ji-Yeon Do, Younghoon Go, Chae-Myung Ha, Je-Yong Choi, Seung Huh, Nam Ho Jeoung, Ki-Up Lee, Hueng-Sik Choi, Yu Wang, Keun-Gyu Park, Robert A. Harris, In-Kyu Lee

**Affiliations:** 1 Department of Biomedical Science, Graduate School of Medicine, Kyungpook National University; 2 Department of Internal Medicine, Kyungpook National University; 3 Leading-edge Research Center for Drug Discovery and Development for Diabetes and Metabolic Disease, Kyungpook National University; 4 New Drug Development Center, Daegu-Gyeongbuk Medical Innovation Foundation; 5 Department of Biochemistry and Cell Biology, Kyungpook National University; 6 Department of Surgery, Kyungpook National University, Daegu, Republic of Korea; 7 Department of Fundamental Medical and Pharmaceutical Sciences, Catholic University of Daegu, Gyeongsan, Republic of Korea; 8 Department of Internal Medicine, Asan Medical Center, University of Ulsan College of Medicine, Seoul, Republic of Korea; 9 National Creative Research Initiatives Center for Nuclear Receptor Signals and Hormone Research Center, School of Biological Sciences and Technology, Chonnam National University, Gwangju, Republic of Korea; 10 State Key Laboratory of Pharmaceutical Biotechnology and Department of Pharmacology and Pharmacy, The University of Hong Kong, Hong Kong, China; 11 Roudebush VA Medical Center and the Department of Biochemistry and Molecular Biology, Indiana University School of Medicine, Indianapolis, IN, USA; 12 Department of Internal Medicine, Soonchunhyang University Gumi Hospital, Gumi, Republic of Korea; 13 GIST College, Gwangju Institute of Science and Technology; 14 Research Institute of Clinical Medicine, Chonnam National University Hwasun Hospital, Gwangju, Republic of Korea; 15 BK21 plus KNU Biomedical Convergence Programs at Kyungpook National University, Daegu, Republic of Korea

## Abstract

Vascular calcification, a pathologic response to defective calcium and phosphate homeostasis, is strongly associated with cardiovascular mortality and morbidity. In this study, we have observed that pyruvate dehydrogenase kinase 4 (PDK4) is upregulated and pyruvate dehydrogenase complex phosphorylation is increased in calcifying vascular smooth muscle cells (VSMCs) and in calcified vessels of patients with atherosclerosis, suggesting that PDK4 plays an important role in vascular calcification. Both genetic and pharmacological inhibition of PDK4 ameliorated the calcification in phosphate-treated VSMCs and aortic rings and in vitamin D_3_-treated mice. PDK4 augmented the osteogenic differentiation of VSMCs by phosphorylating SMAD1/5/8 via direct interaction, which enhances BMP2 signaling. Furthermore, increased expression of PDK4 in phosphate-treated VSMCs induced mitochondrial dysfunction followed by apoptosis. Taken together, our results show that upregulation of PDK4 promotes vascular calcification by increasing osteogenic markers with no adverse effect on bone formation, demonstrating that PDK4 is a therapeutic target for vascular calcification.

Vascular medial calcification is prevalent in patients with diabetes, chronic renal failure (CRF), and in aging. It contributes to the high cardiovascular risk associated with hypertension, left ventricular hypertrophy, and compromised coronary perfusion^1^,^2^,^3^. It results from transdifferentiation of vascular smooth muscle cells (VSMCs) into osteogenic cells in response to bone morphogenetic protein 2 (BMP2) signaling^4^,^5^. Under pro-calcific stimuli such as high inorganic phosphate (Pi) in serum from CRF patients^6^ or in the presence of toxic levels of vitamin D^7^,^8^, BMP2 binds its receptor, BMP2-activated type 2 receptor (BMPR-2), to initiate a signaling cascade^9^. Activation of BMPR-2 triggers formation of a receptor complex that activates the protein kinase BMP2-activated type 1 receptor (BMPR-l)^10^. Activated BMPR-1 phosphorylates small mothers against decapentaplegic (SMAD) 1/5/8, which move into the nucleus where they assemble into a transcriptional machinery that regulates expression of genes that mediate the osteogenic switch^5^,^10^. These genes include Runt-related transcription factor 2 (RUNX2), alkaline phosphatase (ALP), and matrix protein osteocalcin (BGLAP)^11^,^12^,^13^,^14^.

Pyruvate dehydrogenase kinase (PDK) 4 is a key enzyme for regulation of glucose oxidation via inhibition of the pyruvate dehydrogenase complex (PDC)^15^ by phosphorylation of PDHE1α (a subunit of PDC) in mitochondria. High levels of PDK4 are associated with dysregulation of glucose metabolism in diabetes^16^,^17^. In this study, we examined whether upregulation of PDK4 is required for vascular calcification. Several factors that induce vascular calcification also increase PDK4 expression; these include glucocorticoids^18^,^19^, cAMP via protein kinase A^20^,^21^, angiotensin II^22^,^23^, and LXR agonists^24^,^25^. Furthermore, vascular calcification and PDK4 expression are strongly attenuated by insulin^19^,^26^. Since PDK4 regulates PDC in the mitochondrial matrix space^27^ and SMAD1/5/8 are located in the cytosol and nucleus^10^, direct regulation of SMAD1/5/8 by PDK4 has not been considered physically possible. However, under conditions that promote calcification such as high Pi, or toxic levels of vitamin D, extra PDK4 activity might play a role in regulating osteogenic gene expression, in which it promotes vascular calcification by directly phosphorylating SMAD1/5/8, in addition to inducing mitochondrial dysfunction followed by apoptosis. These findings delineate a novel role for PDK4 in vascular calcification and suggest that this enzyme is a promising drug target for the treatment of patients with vascular calcification.

## Results

### PDK4 expression is correlated with osteogenic differentiation in human calcified vessels

**Figure 1:**
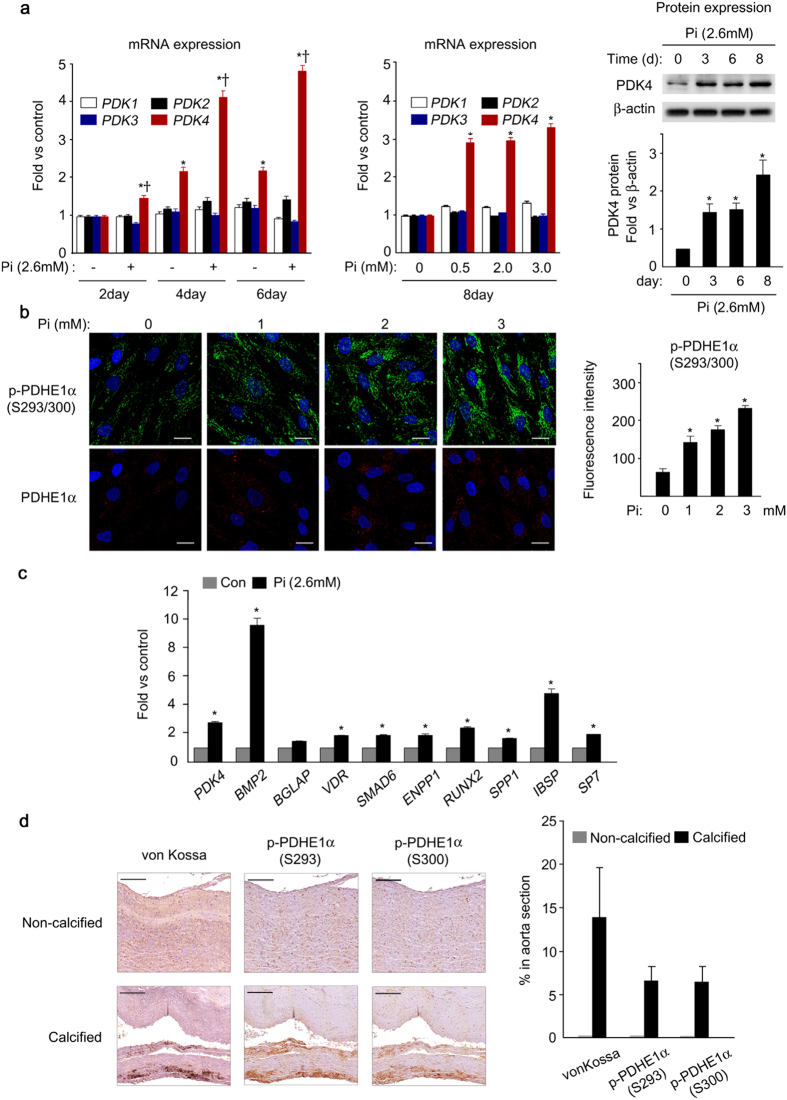
PDK4 is upregulated in the development of vascular calcification. **(a)** mRNA (left) and protein (right) expression of PDK isoenzymes during Pi-induced calcification in human VSMCs in a time-dependent and dose dependent manner (n = 4). *P < 0.05 compared with day 0. ^†^*P* < 0.05 compared with untreated control. **(b)** Immunofluorescence staining and quantification of total PDHE1α and p-PDHE1α during Pi-induced calcification in human VSMCs (n = 4). Scale bar = 20μm. **(c)** Expression of osteogenic genes and PDK4 in Pi-induced human VSMCs on day 3 (n = 3). *P < 0.05 compared with untreated control. **(d)** Representative immunohistochemical results for p-PDHE1α S293/S300 in calcified vessels of patients with vascular calcification, compared to a non-calcified control. Scale bar = 200 μm. Data presented in graphs represent means ± S.E.M.

The expression profile of PDK isoenzymes in vascular calcification was evaluated in human VSMCs treated with high concentrations of Pi to induce calcification. The mRNA and protein levels of PDK4 were markedly increased in a time-dependent manner as well as dose-dependent manner, whereas expression of other PDK isoenzymes (*Pdk 1, 2,* and *3*) was not significantly altered (Fig. 1a). The amount of phosphorylated PDHE1α (p-PDHE1α), a subunit of pyruvate dehydrogenase complex (PDC) and surrogate marker of PDK activity, was also increased in Pi-treated human VSMCs (Fig. 1b and Supplementary Fig. S1). Incubation of VSMCs with Pi also induced expression of mineralization-regulating genes such as *BMP2, RUNX2* (*known as Cbfa1*), *VDR, SP7* (*known as Osterix*), matrix protein such as *BGLAP* (*known as osteocalcin*) and *IBSP* (*known as bone sialoprotein*), and counter-regulatory genes such as *SMAD6 and SPP* (*known as osteopontin*),^9^ as well as upregulation of PDK4 (Fig. 1c).

We next tried to determine whether PDK activity is increased in calcified vessels of patients with atherosclerosis. Because antibodies suitable for immunohistochemistry are not available for the measurement of the PDKs by this technique, we determined the phosphorylation status of PDHE1α as an indirect measure PDK activity. Since the PDKs phosphorylates serines 293 and 300 of PDHE1α^28^, phosphorylation site-specific antibodies were used to examine the level of p-PDHE1α in calcified vessels from 8 patients with atherosclerosis (medical information for these patients is provided in Supplementary Table S1) relative to non-calcified vessels from a healthy donor after brain death. Expression of both p-PDHE1α Ser293 and Ser300 were significantly increased in the calcified lesions, as defined by calcium deposition in calcified vessels from those patients relative to non-calcified regions of the same vessels (Fig. 1d and Supplementary Fig. S2). Since we find that PDK4 is the only PDK that increases under conditions that promote calcification, these findings suggest PDK4 is upregulated by conditions that promote upregulation of osteogenic gene markers and calcification in human vessels and VSMCs.

### Genetic and pharmacological inhibition of PDK4 prevent vascular calcification

**Figure 2:**
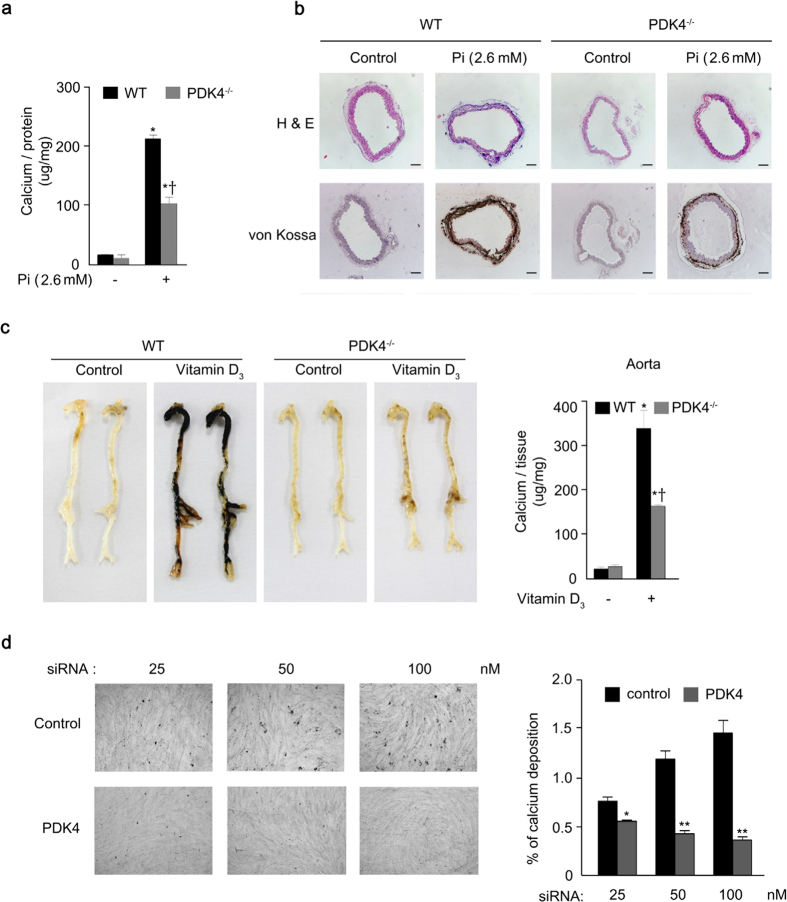
Vascular calcification is attenuated by PDK4 knockdown. **(a)** Calcium deposition in Pi-induced primary VSMCs from WT and PDK4^−/−^ mice (n = 5 per groups). *P < 0.05 compared with untreated control. ^†^*P* < 0.05 compared with WT treated with Pi. **(b)** H&E staining and von Kossa staining of cross-sections of descending aorta rings *ex vivo* cultured with Pi from WT and PDK4^−/−^ mice (representative samples with n = 3 per groups). Scale bar = 100 μm **(c)** Vitamin D_3_-induced calcium deposition (left) and score (right) in aortas from WT and PDK4^−/−^ mice (representative samples with n = 5 per experimental group, n = 3 per control group). *P < 0.05 compared with untreated control. ^†^*P* < 0.05 compared with WT treated with Pi. **(d)** Calcium deposition and quantification by acute PDK4 siRNA knockdown in human VSMCs (n = 3). *P < 0.05, **P < 0.01 compared with the corresponding control. Data presented in graphs represent means ± S.E.M.

To further pursue the possibility that PDK4 plays an important role in vascular calcification, aortic VSMCs from PDK4^−/−^ mice were cultured in media that promote calcification. Calcium deposition was significantly reduced in calcified VSMCs from PDK4^−/−^ mice (Fig. 2a). Similar results were obtained with *ex vivo* aortic rings prepared from WT and PDK4^−/−^ mice based on von Kossa staining after Pi-induced calcification (Fig. 2b). To evaluate the effect of PDK4 deletion in an *in vivo* model, mice were treated with high doses of vitamin D_3_, which has been shown to induce medial calcification in different animal models^7^,^29^,^30^. WT mice exhibited remarkable calcification in the aortas after vitamin D_3_ treatment, which was significantly reduced in PDK4^−/−^ mice (Fig. 2c). (Additional physiological parameters for vitamin D_3_-induced calcification mouse models are shown in Supplementary Figure S3a–c.) We confirmed that Pi-induced calcification in VSMCs was significantly attenuated by siRNA-mediated PDK4 mRNA knockdown (80 ± 3% of control with 100 nM siRNA) (Fig. 2d).

**Figure 3:**
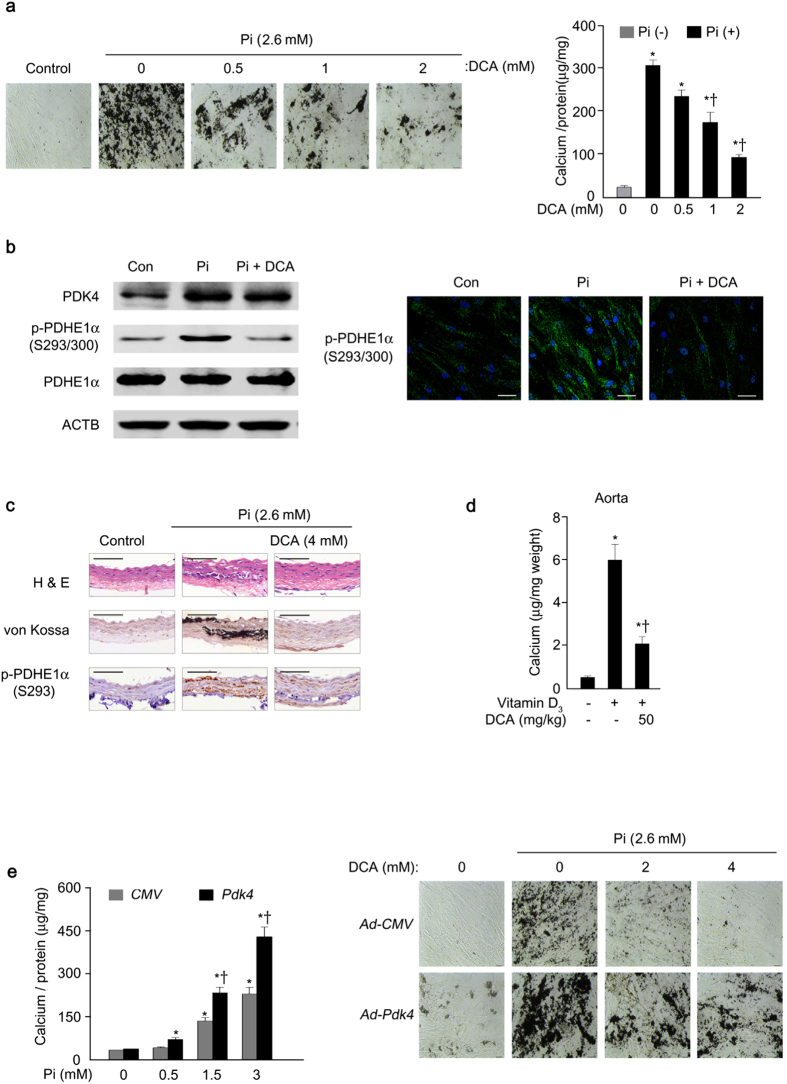
Vascular calcification is suppressed by dichloroacetate (DCA), a pharmacological inhibitor of PDK. **(a)** Calcium deposition (left) and score (right) in Pi-induced human VSMCs in the presence of the indicated concentrations of DCA (n = 3). *P < 0.05 compared with Pi-untreated control. ^†^*P* < 0.05 compared with Pi-treated control. **(b)** Western blot analysis (left) and immunofluorescence analysis (right, green) for p-PDHE1α S293/S300 in human VSMCs on day 3 after Pi treatment with or without DCA. Nuclei were stained with DAPI (blue) (n = 4). Scale bar = 50 μm. **(c)** H&E staining, calcium deposition, and corresponding p-PDHE1α S293 of cross-sections of Pi-treated rat common carotid arteries *ex vivo* with or without DCA (n = 5). Scale bar = 100 μm. **(d)** Vitamin D_3_-induced calcium deposition in aortas of DCA-treated and –untreated WT mice (n = 5 mice per group). *P < 0.05 compared with Vitamin D_3_-untreated control. ^†^*P* < 0.05 compared with Vitamin D_3-_treated control. **(e)** Calcium score measured in human VSMCs infected with Ad-*Pdk4* compared to control (left) and calcium deposition by *PDK4* overexpression (Ad-*Pdk4*) with different concentrations of DCA co-treatment (right) (n = 3). *P < 0.05 compared with Pi-untreated control. ^†^*P* < 0.05 compared with Ad-*CMV*. Data presented in graphs represent means ± S.E.M.

Concomitantly, DCA^31^, a general pharmacological inhibitor of the PDK isoforms, attenuated the calcification of VSMCs in a dose-dependent manner as assessed by von Kossa staining and calcium levels (Fig. 3a) and decreased Pi-induced p-PDHE1α Ser 293/300 levels (Fig. 3b and Supplementary Fig. S3d). DCA attenuated the calcification of rat aortic rings *ex vivo* (Fig. 3c and Supplementary Fig. S3e) and vascular calcification in vitamin D_3_-treated mice *in vivo* (Fig. 3d). The experimental schedule of DCA treatment in vitamin D_3_-treated mice is shown in supplementary Figure S3f,g. Overexpression of PDK4 by adenoviral delivery-*Pdk4* (Ad*-Pdk4*) in human VSMCs significantly increased calcification in a dose-dependent manner that was effectively attenuated by DCA (Fig. 3e). Taken together, the findings with DCA suggest that PDK4 is a promising target for the treatment of vascular calcification.

### PDK4 augments the osteogenic switch in VSMCs

**Figure 4:**
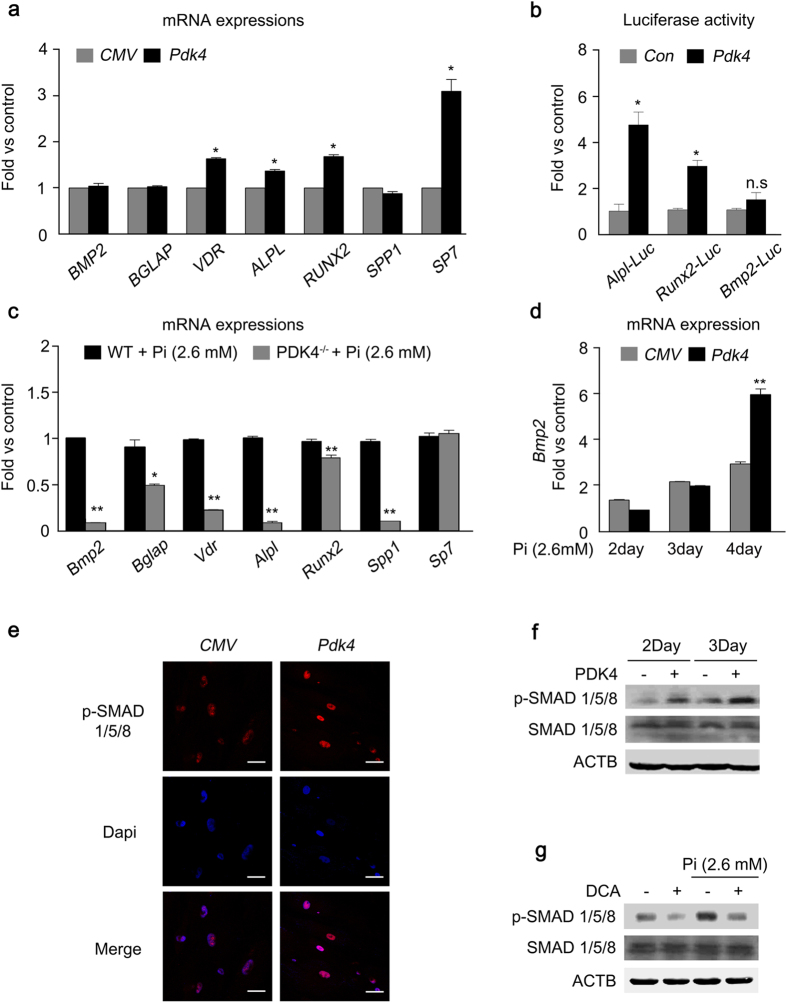
The levels of PDK4 are positively correlated with osteogenic markers. **(a)** mRNA levels of osteogenic genes measured in human VSMCs after infection with Ad-*Pdk4* (n = 3). *P < 0.005 compared with Ad-*CMV*. **(b)** Luciferase activity measured in HEK293 cells after transfection with a *Pdk4* vector and luciferase reporter constructs for fragments of *Runx2* (−4615/+60), *Alpl* (1.9 kb), and *Bmp2* (−2712/+165) (n = 3). *P < 0.05 compared with *Con* (pc DNA) vector. n.s, non-significance. **(c)** RT-PCR analysis of osteogenic genes in VSMCs from WT and PDK4^−/−^ mice cultured with Pi (n = 3). *P < 0.05, **P < 0.01 compared with WT. **(d)** RT-PCR analysis of *Bmp2* gene expression in human VSMCs infected with Ad-*CMV* or Ad-*Pdk4* (n = 3). **P < 0.01 compared with Ad-*CMV*. **(e)** Immunostaining for p-SMAD1/5/8 in human VSMCs after Ad-*CMV* or Ad-*Pdk4* infection. p-SMAD1/5/8 (red), DAPI for nuclei (blue). Scale bar = 50 μm. **(f,g)** Western blots of p-SMAD 1/5/8 and total SMAD 1/5/8 in human VSMCs infected with Ad-*CMV* or Ad-*Pdk4* (**f**) and Effect of DCA on p-SMAD1/5/8 during calcification (**g**). Data presented in graphs represent means ± S.E.M.

Upregulation of osteogenic gene expression is a key cellular process in vascular calcification^5^. PDK4 overexpression by Ad*-Pdk4* in human VSMCs increased both mRNA expression of osteogenic genes and increased promoter activity of *Runx2* and *Alpl* in a dose-dependent manner, with no change of *Bmp2* mRNA expression (Fig. 4a,b and Supplementary Fig. S4a). The increased expression of *Alpl* mRNA by *Pdk4* overexpression was reversed by DCA treatment (Supplementary Fig. S4b). Likewise, master osteogenic regulators were significantly reduced in Pi-treated VSMCs obtained from PDK4^−/−^ mice compared to WT mice (Fig. 4c). Interestingly, overexpression of PDK4 did not alter BMP2 expression, in contrast to the increase in BMP2 expression caused by incubating human VSMCs in Pi-containing media (Fig. 4a,b and Fig. 1d). Because Bmp2 promoter activity did not increase with PDK4 overexpression (Fig. 4b), we tested whether Bmp2 expression was altered in VSMCs from PDK4^-/-^ mice under physiological condition. The expression of Bmp2 did not change in these cells in the absence of Pi (Supplementary Fig. S4c). Under Pi-treated condition, we observed that Bmp2 expression was only increased by PDK4 overexpression at late time points (4days) (Fig. 4d), suggesting that PDK4 may indirectly affect BMP2 expression only after Pi-treatment.

Since PDK4 upregulated expression of several osteogenic genes which are induced by BMP2, we postulated that PDK4 may be involved in BMP2 downstream signaling without affecting the expression and promoter activity of BMP2 itself. We tested whether PDK4 alters the phosphorylation state of SMAD1/5/8, downstream components of the BMP2 signaling pathway that, when phosphorylated, translocate into the nucleus to activate expression of osteogenic genes^32^. The immunofluorescence analysis data showed that PDK4 overexpression increased nuclear translocation of p-SMAD in human VSMCs, consistent with increased p-SMAD protein expression (Fig. 4e,f). The enhanced p-SMAD 1/5/8 expression in calcifying media was suppressed by DCA treatment (Fig. 4g).

**Figure 5:**
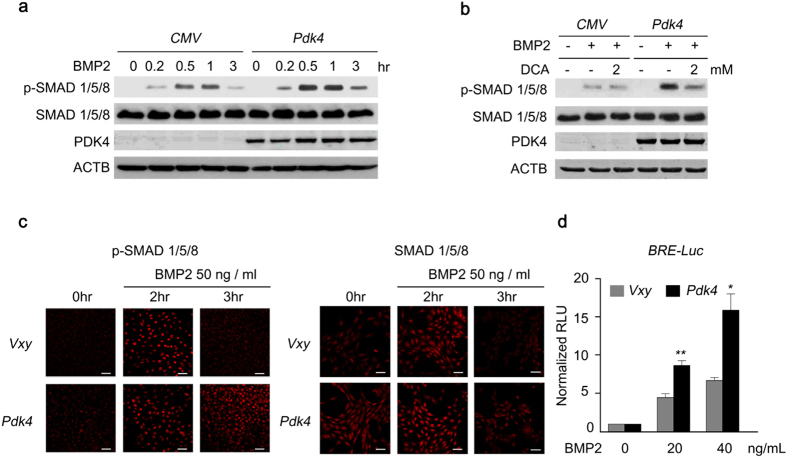
The level of p-SMAD is augmented by PDK4 activity in BMP induction. (**a, b**) Immunoblot analysis of p-SMAD1/5/8 and SMAD1/5/8 in human VSMCs infected with Ad-*CMV* or Ad-*Pdk4* under BMP2 (20 ng/ml) stimulation for indicated number of hours (**a**) with indicated concentrations of DCA (**b**) (n = 3). **(c)** Immunostaining for p-SMAD1/5/8 (left) and SMAD1/5/8 (right) in C2C12 cells infected with control retrovirus (*Vxy*) or retrovirus expressing *Pdk4* after stimulation with BMP2 (50 ng/ml) (n = 3). Scale bar = 50 μm. **(d)** C2C12 cells infected with control retrovirus or *Pdk4* expressing retrovirus were transfected with a BMP/luciferase reporter (BRE-*Luc*) containing *Bmp2* response element (12X*GCGC*). Cells were stimulated for 48 h with BMP2 (n = 3). *P < 0.05, **P < 0.01 compared with control (*Vxy*). Data presented in graphs represent means ± S.E.M.

Next, we investigated the underlying mechanism by which PDK4 upregulated osteogenic genes induced by BMP2. We confirmed that the phosphorylation level of SMAD1/5/8 was consistently upregulated by PDK4 after BMP2 induction, which was diminished by DCA treatment (Fig. 5a,b). Likewise, PDK4 overexpression augmented the levels of p-SMAD 1/5/8 after BMP2 induction in C2C12 cells and human VSMCs (Fig. 5c and Supplementary Fig. S4d). To monitor the effect of PDK4 overexpression on SMAD1/5/8 transcriptional activity, C2C12 cells were transfected with a vector carrying a luciferase reporter under the control of a BMP response element, the sequence that SMAD1/5/8 bind to promote transcription by BMP2 (Fig. 5d). As expected, overexpression of PDK4 in C2C12 cells resulted in increases in p-SMAD1/5/8 levels after BMP2 induction and potentiated the transcriptional activity of BMP2 (Fig. 5c,d). This data demonstrates that an increase in p-SMAD1/5/8 mediated by PDK4 plays an important role in regulating osteogenic gene expression under calcifying conditions.

### PDK4 directly binds to and phosphorylates SMAD1/5/8

**Figure 6:**
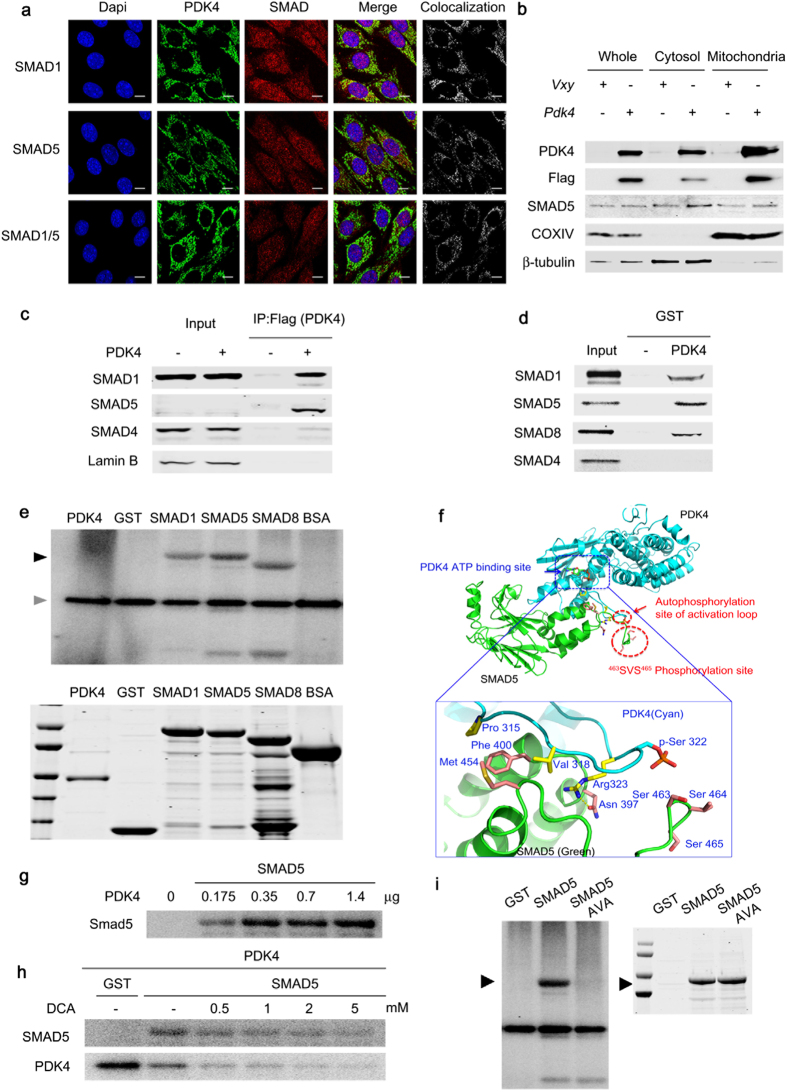
PDK4 directly interacts with SMAD1/5/8. (**a**) Immuno-fluorescence images of C2C12 cells infected with control retrovirus or *Pdk4*-expressing retrovirus. The sections were co-stained with antibodies for PDK4 (green), SMAD1, SMAD5, SMAD1/5 (red), and DAPI (blue). Colocalization was confirmed by a LSM5 ZEISS exciter (white). Scale bar = 10 μm. **(b)** Western blot for SMAD5 and PDK4 in mitochondrial and cytosolic extracts from C2C12 cells infected with control retrovirus or *Pdk4* expressing retrovirus (n = 3). **(c)** Co-immunoprecipitation analysis of C2C12 infected with control (Vxy) or C-terminal Flag epitope-tagged *Pdk4*-expressing retrovirus. (**d**) GST pull-down assays with GST-PDK4 and ^35^S-labelled SMADs. **(e)** *In vitro* kinase assay with [γ-^32^P]-ATP showing phosphorylation of SMAD1, SMAD5, and SMAD8 by PDK4 (black arrow), auto-phosphorylation of PDK4 (positive control, gray arrow). **(f)** Structure osf the complex showing the S463 and S465 phosphorylation sites of the SMAD5 and PDK4 complex. (**g**) *In vitro* kinase assay showing the phosphorylation of SMAD5 by indicated doses of PDK4 proteins. (**h**) *In vitro* kinase assay showing that DCA inhibits the phosphorylation of SMAD5 and auto-phosphorylation induced by PDK4. (**i**) The elimination of SMAD5 phosphorylation by mutation of S463 and S465 of SMAD5 to Ala (SMAD5 AVA). Data presented in graphs represent means ± S.E.M.

Because PDK4 and SMAD1/5/8 are located in different subcellular compartments, a direct interaction between PDK4 and SMAD seems unlikely. Conversely, it has been reported that SMAD5 is present in the mitochondria of a human chondrogenic cell line^33^, and the mammalian PDK4 orthologue in *Caenorhabditis elegans* is dual-localized in the cytosol and mitochondria^34^. Therefore, these reports raised the possibility of a direct interaction between PDK4 and SMADs. Furthermore, the MitoProt ІІ algorithm program predicts with high probabilities (86.5% to 91.7%) that mouse and human SMAD1/5/8 have mitochondrial targeting sequences of 24 to 30 amino acids. Because transfection efficiency is poor with human primary VSMCs, co-localization studies in these cells are not feasible. Therefore, confocal imagining analysis of the subcellular localization of PDK4 and SMAD proteins was conducted with C2C12 cells, which like VSMCs increase PDK4 expression and undergo calcification in a high Pi media (Supplementary Fig. S5a,b). As expected, overexpressed PDK4 was predominantly localized in mitochondria and partially in the cytosol whereas SMAD 1 and 5 were mainly localized in the cytosol and partially in the mitochondria (Supplementary Fig. S6a). Furthermore, immunostaining using confocal microscopy revealed colocalization of PDK4 and SMAD1/5 in the cytosol of PDK4-overexpressing C2C12 cells (Fig. 6a). When we performed immunoblots of PDK4 and SMAD5 with cytosolic and mitochondrial fractions of C2C12 cells expressing *Pdk4,* SMAD5 and PDK4 were detected in both the mitochondrial and the cytosolic fraction (Fig. 6b). These data showed the possibility of direct interaction between PDK4 and SMAD1/5/8 in the cytoplasm as well as mitochondria.

We further investigated this potential interaction by co-immunoprecipitation from PDK4-expressing C2C12 cells. PDK4 bound tightly to SMAD1 and SMAD5 but not SMAD4 (Fig. 6c). To confirm the physical interaction, SMAD proteins were prepared by *in vitro* transcription/translation and incubated with a GST-PDK4 fusion protein. SMAD1, SMAD5, and SMAD8, but not SMAD4, were pulled down by GST-PDK4 (Fig. 6d). The experiment using PDK4 prepared by *in vitro* transcription/translation and immobilized GST-SMADs further confirmed that PDK4 directly interacts with SMAD1, SMAD5, and SMAD8, but not with SMAD4 (Supplementary Fig. S6b). Based on an *in vitro* kinase assay, PDK4 directly phosphorylated SMAD1/5/8 (Fig. 6e). To validate the interaction between PDK4 and SMAD, a protein-protein interaction model was developed based on the structure of ADP-bound PDK4. This model predicted that residues Asn397, Phe400, and Met454 located within the ATP-lid of PDK4 interact with residues Arg323, Pro315, and Val318 that surround the Ser463 and Ser465 in SMAD5 that are phosphorylated by BMP receptor 1 (Fig. 6f). Considerable amino acid sequence identity (31% of 45 residues) and similarity (additional 29%) exists in the ATP-binding domains of PDK4 and BMP receptor 1^35^. Phosphorylation of SMAD5 was markedly increased by PDK4 in a dose-dependent manner (Fig. 6g), and DCA decreased this activity of PDK4 (Fig. 6h). DCA also inhibited auto-phosphorylation of PDK4 (Fig. 6h) by a direct inhibitory effect upon PDK4^36^. SMAD5 also inhibited auto-phosphorylation of PDK4 (Fig. 6h), consistent with SMAD5 being a better substrate than PDK4 itself. Phosphorylation of SMAD5 by PDK4 was completely blocked by replacing Ser463 and Ser465 of SMAD5 with alanine residues (sites that are phosphorylated by BMPR-1) (Fig. 6i). Taken together, these findings indicate that PDK4 binds SMAD1/5/8 and phosphorylates SMAD5 (and most likely SMAD1/8) on the same residues that are phosphorylated by BMPR-1.

### PDK4 exacerbates apoptosis and mitochondrial dysfunction in calcified VSMCs

**Figure 7:**
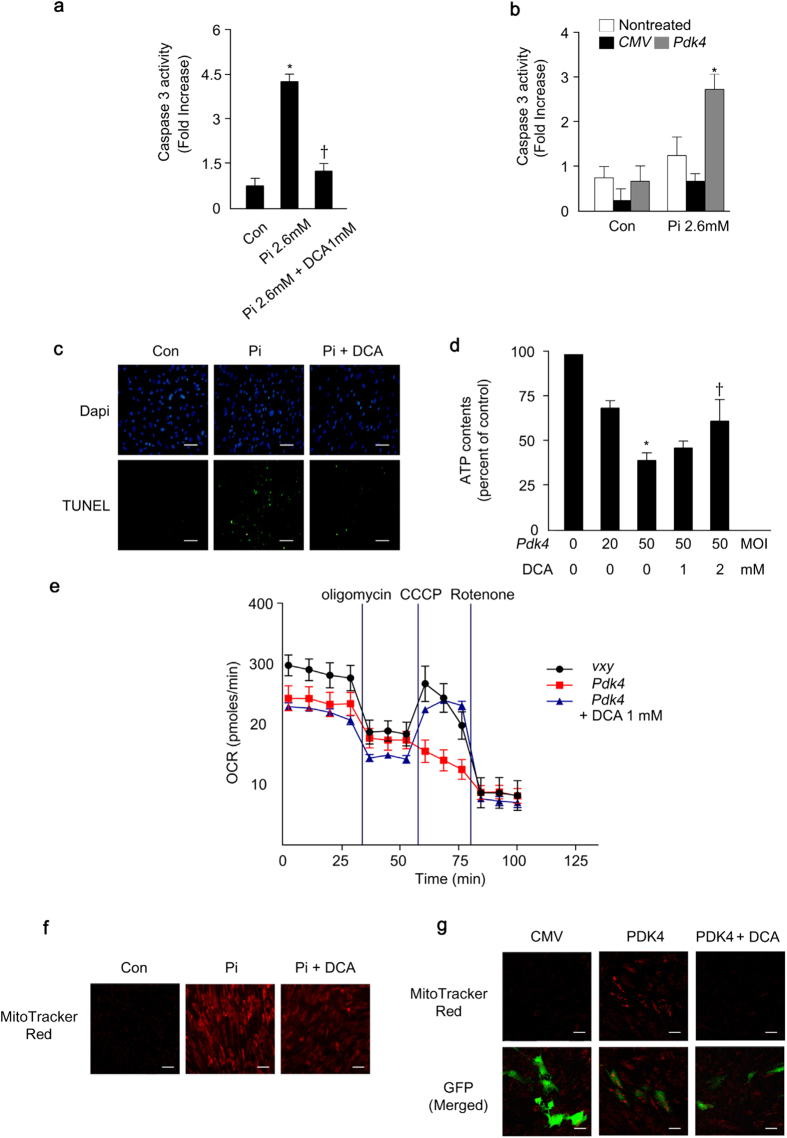
PDK4 exacerbates apoptosis and mitochondrial dysfunction in calcified VSMCs. **(a)** Caspase 3 activity in Pi-treated human VSMCs with or without DCA (n = 4). *P < 0.05 compared with untreated control. ^†^*P* < 0.01 compared with Pi-treated control. **(b)** Caspase 3 activity in human VSMCs infected with Ad-*CMV* or Ad-*Pdk4* with or without Pi treatment (n = 5). *P < 0.05 compared with Ad-*CMV* **(c)** TUNEL staining in human VSMCs during calcification with or without DCA. Scale bar = 100 μm. **(d)** ATP contents in human VSMCs infected with Ad-*CMV* and Ad-*Pdk4* with different DCA concentrations (n = 3). *P < 0.05 compared with Ad*-CMV*. ^†^*P* < 0.05 compared with Ad-*Pdk4* 50 MOI. (**e**) Oxygen consumption ratio in C2C12 cells infected *PDK4* expressing retrovirus and *Vxy* and DCA effect. After basal OCR determination, cells were treated with oligomycin, CCCP, and rotenone in these cells. DCA (1 mM) was treated in these cells (n = 5). (**f**) Mitochondrial ROS (MitoTracker Red) in human VSMCs during calcification induced by Pi and DCA treatment. (**g**) Mitochondrial ROS (MitoTracker Red) in human VSMCs infected *Pdk4* expressing retrovirus and *Vxy* after DCA treatment. (n = 3). Scale bar = 50 μm. Data presented in graphs represent means ± S.E.M.

The oxidative stress and consequent apoptosis in VSMCs play a critical role in the pathogenesis of vascular calcification^37^. In addition, the primary role of PDK4 is as an important regulator in mitochondria for transforming pyruvate into acetyl-CoA by PDC. Previously, we reported that α-lipoic acid, which also inhibits PDK4^38^, attenuates vascular calcification and Pi-induced apoptosis of VSMCs by improving mitochondrial function and the intracellular redox status^39^. Therefore, we evaluated the effects of PDK4 on mitochondrial function and reactive oxygen species (ROS) generation in VSMCs. As shown in Fig. 7a, caspase-3 activity increased in human VSMCs after Pi treatment and DCA reversed the increase. Likewise, PDK4 significantly increased caspase-3 activity under high-Pi media (Fig. 7b). According to the TUNEL assay, Pi-stimulated apoptosis was blunted by the PDK pharmacological inhibitor DCA (Fig. 7c).

To determine the effects of PDK4 on mitochondrial respiratory function, we measured ATP contents and oxygen consumption rate (OCR). Ad-*Pdk4* infected human VSMCs induced mitochondrial dysfunction, demonstrated by decreased ATP content, whereas this was ameliorated by DCA treatment (Fig. 7d). Basal oxygen consumption rate (OCR) and maximal respiration capacity were also reduced in C2C12 cells that were infected with retrovirus expressing *Pdk4* (Fig. 7e). DCA reversed the negative effects of PDK4 overexpression (Fig. 7e). These results indicate that PDK4 reduces mitochondria function which may contribute to the pathogenesis in vascular calcification.

In addition, DCA treatment blocked Pi-induced mitochondrial ROS generation (Fig. 7f), a well-known pro-apoptotic factor that promotes calcification of VSMCs. Moreover, Ad-*Pdk4* infected human VSMCs increased mitochondria ROS generation (Fig. 7g) and DCA treatment blocked PDK4 induced mitochondrial ROS generation (Fig. 7g). These findings suggest that PDK4 exacerbates vascular calcification not only by promoting BMP-SMAD signaling but also by inducing mitochondrial-dependent apoptosis and mitochondrial dysfunction.

### PDK4 deficiency does not adversely affect bone remodeling

**Figure 8:**
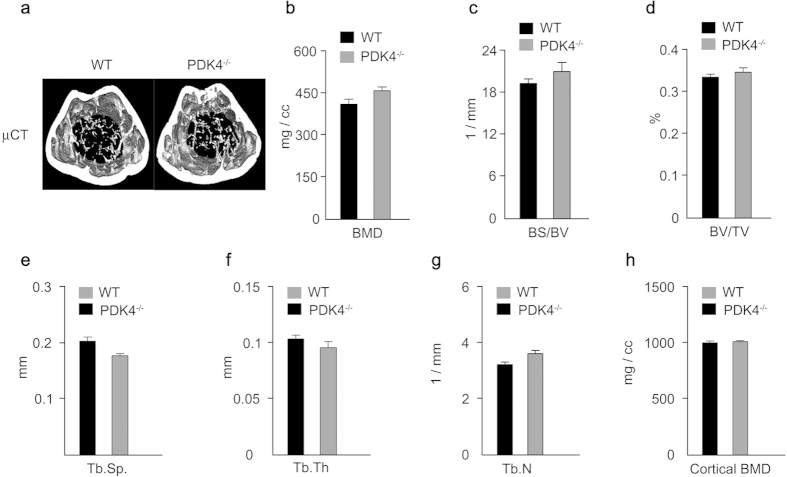
PDK4 knockdown does not adversely affect normal bone remodeling. **(a)** Representative Micro-CT image of distal femurs from WT and PDK4^−/−^ mice. **(b–h)** Bone morphometric parameters of the femurs from WT and PDK4^−/−^ mice. (**b**) Trabecular bone mineral density (BMD), (**c**) Bone surface/bone volume (BS/BV; spatial distribution of trabeculae), (**d**) Bone volume/tissue volume (BV/TV: amount of trabecular bone within the cancellous space), (**e**) Trabecular separation (Tb. Sp), (**f**) Trabecular thickness (Tb. Th), (**g**) Trabecular number, (**h**) Cortical BMD from WT and PDK4^−/−^ mice (n = 5 per group). Data presented in graphs represent means ± S.E.M.

The processes involved in vascular calcification and osteoblast differentiation for bone formation are similar^29^,^40^,^39^. Therefore, we examined whether PDK4 affects bone remodeling by two types of pre-osteoblasts, MC3T3E1 and bone marrow stromal cells (BMSCs) derived from PDK4^−/−^ and WT mice. Osteoblast differentiation and the expression of osteoblast marker genes such as *Alpl, Runx2*, and *Tnfrsf11b* (also known as *Opg*) decreased in MC3T3E1 cells infected with Ad-*Pdk4*, consistent with von Kossa staining and ALP activity (Supplementary Fig. S7a–c). Likewise, mineralization detected by von Kossa staining, ALP activity, and expression of osteogenic gene markers were increased in bone marrow stem cells (BMSCs) from PDK4^−/−^ mice compared to WT (Supplemental Fig. S7d–f), indicating that PDK4 induced opposite effects in VSMCs and pre-osteoblasts. Although PDK4 deletion produced positive effects on osteoblastogenesis, consistent with a previous report^41^, the micro-computed tomography (micro-CT) of the femurs from PDK4^−/−^ and WT mice at 17 weeks of age showed no differences in parameters including bone mineral density, bone surface/bone volume, bone volume/tissue volume ratios, trabecular separation, trabecular thickness, trabecular number, and cortical BMD (Fig. 8). Thus, deletion of PDK4 effectively ameliorated vascular calcification without adverse effects on bone remodeling.

## Discussion

This study provides the first evidence that PDK4 plays an important role in osteogenic switching in VSMCs by direct phosphorylation of SMAD1/5/8 under calcifying conditions. Concomitantly, we found that p-PDHE1α Ser293 and Ser300 are increased in calcified regions of the vessels from patients with vascular diseases, suggesting that enhanced PDK activity presumably due to increased PDK4 expression occurs during vascular calcification. On a molecular basis, we propose that PDK4 activates SMAD1/5/8 by phosphorylation, which leads to translocation of p-SMAD into the nucleus for transcriptionally regulating osteogenic markers in a similar manner to the BMP2 signaling pathway. Accordingly, significant similarity exists in the primary amino acid sequences of the ATP-binding and kinase domains of PDK4 and BMPR-1, and the serine phosphorylated by PDK4 at site 1 in PDHE1α is in the same sequence context (SXS) as the motif phosphorylated in SMAD1/5/8 by BMPR-1. A unique kinetic property of PDK4 favors its ability to phosphorylate SMAD1/5/8. In the free form the basal activities of the other PDK isoforms is low and only becomes significant upon binding to the core of the PDC. In contrast, the free form of PDK4 exhibits robust enzyme activity towards suitable substrates in the absence of the PDC^36^.

Interestingly, PDK4 overexpression by itself does not induce vascular calcification mediated by the phosphorylation of SMAD, demonstrating that additional modification of PDK4 by canonical BMP signaling might be necessary for kinetic activity of SMAD under calcification conditions. Since PDK4 is located in the mitochondrial matrix, phosphorylation of SMAD1/5/8 by PDK4 is unexpected. Although this dilemma was not fully resolved in this study, findings to support our data have been reported. Chen *et al.* provided evidence that pyruvate dehydrogenase phosphatase (PDP) 1, the counterpart of PDK4 for regulation of PDC activity, dephosphorylates SMAD1 directly in vertebrates and the analogous protein MAD (mothers against decapentaplegic in *Drosophila*), even though PDP1 was believed to be located exclusively in mitochondria^42^. Consistently, the phosphorylation state of DAF8 in *C. elegans*, the orthologue of vertebrate SMAD 1/5/8, is also regulated by PDP1^43^. These observations suggest PDP is a dual-localized cytosolic/mitochondrial enzyme. Our immunohistochemical data and western blot analysis indicate that both PDK4 and SMAD can localize in both the cytosol and the mitochondria during calcification. This phenomenon is supported by recent results showing that PDHK2, the orthologue of PDK4, activates SKN-1, the orthologue of Nrf2, by phosphorylation in the cytosol of *C. elegans*, suggesting that PDK is also dual-localized in the cytosol and mitochondria^34^,^44^. Consistent with our data, PDHK2 is found in the mitochondria dominantly at very low levels in well-nourished states, and is partially distributed to the cytosol by starvation of *C. elegans*^34^. Furthermore, it has been reported that PDK4 directly binds and regulates the cAMP-response element-binding protein (CREB), which is known to be located in the cytosol^45^. Taken together, these results suggest that that PDP1 and PDK4 are dual-localized cytosolic/mitochondrial enzymes that participate in the regulation of the phosphorylation state of SMAD1/5/8 in humans under pathological conditions.

Since PDK4 plays a prohibitory role in glucose oxidation associated with mitochondrial dysfunction, increased caspase activity and decreased ATP generation followed by apoptosis during calcification were ameliorated by DCA treatment. The recent observation that a shift toward glycolytic breakdown of glucose occurs during vascular calcification^46^ is consistent with high expression of PDK4 and decreased PDC activity found in the present study. We have also observed that glucose consumption and lactate production are increased in Pi-induced VSMC calcification (data not shown). Further studies will be required to ascertain the changes in metabolism in VSMCs during vascular calcification and its relationship with PDK4.

Intensive efforts have been made to identify therapeutic targets for the prevention of vascular calcification because similar processes and key factors are involved in osteogenesis in both vascular calcification and physiologic bone formation. The inhibitory effect of vascular calcification might adversely affect bone remodeling^14^,^47^,^48^. In this study, VSMCs under calcifying media and *ex vivo* ring calcification as well as vitamin D_3_-induced aortic calcification *in vivo* were significantly attenuated in PDK4-deficient mice. Even though PDK4 promotes osteogenic switching in VSMCs under calcifying stimuli, bone remodeling and osteoblastic differentiation in pre-osteoblasts was not adversely affected by PDK4 deficiency. Instead, consistent with our data, PDK4^−/−^ mice are protected from bone loss induced by unloading^41^. Last, a number of clinical and experimental studies have shown an increase in the incidence of vascular calcification and osteoporosis^49^,^50^, further highlighting the potential of PDK4 as a therapeutic target for the treatment of vascular calcification without adverse effects on bone formation.

## Methods

### Human Tissues

The study protocol was approved by the institutional review board and the Regional Board of the Ethics Committee of the Kyungpook National University Hospital (IRB 2012-10-022) and informed consents were obtained from the subjects. All experiments were performed in accordance with approved guidelines of Kyungpook National University. Blood vessels were obtained from 8 patients who underwent vasculoplasty due to atherosclerosis at Kyungpook National University Hospital, Daegu, South Korea. Detailed medical information for the patients is provided in Online Table І. Human VSMCs and healthy vessels for immunohistochemistry were prepared from thoracic aortas of kidney transplantation donors, obtained using a modified explants method as described in the Data Supplement, and were used for experiments at passages 5–7.

### Mouse model of vitamin D_3_-induced aortic calcification

All experiments were approved by the Institutional Animal Care and Use Committee of Kyungpook National University (KNU 2012-83). All experiments were performed in accordance with approved guidelines of Kyungpook National University. A vitamin D_3_-induced aortic calcification mouse model is well established in this laboratory^51^. Six-week-old C57BL/6N male mice were subjected to gavage daily with sterile water as control or with dichloracetate (DCA; Sigma-Aldrich, St. Louis, MO, USA, Cat # D54702) (50 mg kg^−1^) for 13 days. At day 3 after the initial treatment with DCA, the mice were subcutaneously administered vitamin D_3_ (cholecalciferol; Sigma-Aldrich, Cat # C9756, 5.5 × 10^5^ IU kg^−1^ day^−1^) daily for 3 days. Seven days after the final exposure to vitamin D_3_, mice were anesthetized with pentobarbital (50 mg kg^−1^) and whole aortas were collected by surgical dissection to determine calcium content. The colony of PDK4-deficient (PDK4^−/−^) mice^52^ was established in an in-house animal facility. Six-week-old C57BL/6J WT and PDK4^−/−^ male mice were subcutaneously administered vitamin D_3_ (C57BL/6J; 4.5 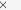
10^5^ IU kg^−1^ day^−1^) daily for 3 days. Thirteen days after the final exposure to vitamin D_3_, mice were anesthetized as described before. Animals were maintained on a 12-h light/12-h dark cycle. All animals used in the experiments were age- and sex-matched and littermates.

### Immunohistochemistry

The vessels were routinely processed for paraffin embedding at a thickness of 10 μm after 4% paraformaldehyde fixation for the analysis. For details of immunohistochemistry, refer to the supplementary material method.

### Induction of *in vitro* VSMC calcification

VSMCs were seeded at a density of 1.3 × 10^4^ cells per cm^2^ and maintained for 2 days until sub-confluent. Calcification was induced by DMEM (high glucose) supplemented with 10% FBS and an additional 2.6 mM Pi (a mixture of Na_2_HPO_4_ and NaH_2_PO_4_, pH 7.4) that borough the final Pi concentration to 3.5 mM). The calcification medium was changed every 2 days for 8 days.

### Antibodies

Anti-p-PDHE1α (S293, S300) (Calbiochem, San Diego, CA, USA, AP1062-1064, respectively) were used for immunohistochemistry. Anti-p-PDHE1α (S293, S300) (Abfrontier), anti-SMAD1/5/8 (Santa Cruz Biotechnology, Dallas, TX, USA, sc-6031-R), anti-p-SMAD 1/5/8 (Cell Signaling Technology, Danvers, MA, USA, #9511), COXIV (Abcam, Cambridge, UK, ab16056) and anti-Flag (Sigma-Aldrich, F1804) antibodies were used for both immunofluorescence and western blotting. Anti-SMAD1 (Invitrogen, Carlsbad, CA, USA, #38-5400), anti-SMAD5 (Abgent, San Diego, CA, USA, AJ1726a), and anti-SMAD1/5 (Santa Cruz Biotechnology, sc-6201) were used for immunofluorescence. Anti-α-tubulin (Applied Biological Materials, G098), anti-lamin B (Santa Cruz Biotechnology, sc-6216), anti-SMAD4 (Cell Signaling Technology, #9515) and anti-PDK4 serum (Abfrontier) were used for western blotting.

### GST tagged protein and *in vitro* kinase assay

Full-length human *SMAD1*, mouse *Smad5*, and rat *Smad8* were inserted into the EcoR1 and Xho1 sites of the pGEX4T-1 vector for expression as a glutathione-*S*-transferase (GST) fusion protein and amplified in *Escherichia coli* BL21. The GST-tagged human SMAD1, mouse SMAD5, rat SMAD8, human SMAD4, and mouse PDK4 proteins were purified for an *in vitro* kinase assay by incubation with glutathione-Sepharose TM4B (GE Healthcare, Sweden) at room temperature for 20 min, followed by extensive washing of the beads with PBS. [^35^S]-Methionine-labeled mouse PDK, human SMAD1, mouse SMAD5, and rat SMAD8 proteins were synthesized *in vitro* using a TnT Quick Coupled Transcription/Translation System (Promega, Madison, WI, USA). The washed glutathione-Sepharose beads were incubated with [^35^S]-methionine-labeled mouse PDK4, human SMAD1, mouse SMAD5, or rat SMAD8 in binding buffer (25 mM HEPES, pH 7.6, 1 mM DTT, 100 mM NaCl, 0.2 mM EDTA, 1.5% BSA, 20% glycerol) at 4 °C for 12 h. Sepharose beads were washed twice with PBS and boiled with SDS-PAGE sample buffer, and the supernatant proteins were resolved in SDS-PAGE gels.

### Binding model prediction of PDK4 and SMAD5

The structure of PDK4 in complex with ADP was obtained from the Protein Data Bank (http://www.pdb.org, pdb code: 2E0A). Discovery Studio3.5 (http://www.accelrys.com) with MODELLER was used for the homology model building of SMAD5. For details of binding model prediction of PDK4 and SMAD5, refer to the supplementary material method.

### Cell culture and transient transfection

Mouse VSMCs were isolated from the thoracic aortas of 5 week old male C57BL/6J mice. Cells were used for experiments after 4 to 5 passages. Human VSMCs were isolated from the thoracic aortas of kidney transplantation donors. For details of cell culture, refer to the supplementary material method.

### Immunohistochemistry

Ethics approval was obtained from the Institutional Review Board of the Kyungpook National University Hospital for all studies with human tissues and the collection of medical data. Informed consent was obtained for all studies with human tissues. The section after fixation by PFA and embedding by paraffin were used for immunohistochemistry analysis. Tissue sections were deparaffinized followed by the retrievation of antigens using steamer (IHCWORLD, IW-1102) at 95 °C for 40 min in 0.01 M citrate buffer, pH 6.0. For the visualization of p-PDHE1α S293 and S300 in arteries, the sections were incubated with antibodies against p-PDHE1α S293 and S300 overnight at 4 ^o^C before detection with rabbit IgG and mouse IgG antibody by UltraVision LP Detection System (Thermo Scientific). Nuclei were counterstained with Mayer’s Hematoxylin (Lillie’s Modification).

### Quantification of Calcium Deposition

For measuring calcium deposited in the extracellular matrix of the cells calcium contents in the supernatant were determined colorimetrically by the *o*-cresolphthaleincomplexone method (Bioassay System, Cat.DICA500). For details of quantification of calcium deposition, refer to the supplementary material method.

### Immunofluorescence analysis

Human VSMC attached on glass in a 6-well plate at a density of 2 × 10^5^ per well, grew to confluence, and were further incubated in the absence or presence of extra Pi for the indicated times. The cells were fixed with 4% PFA for 15 min and permeabilized with 0.1% Triton X-100 for 15 min at room temperature. The permeabilized cells were incubated with antibody for PDHE1α, SMAD1, SMAD5, SMAD1/5 and p-SMAD1/5/8 in zymogen Ab diluents solution overnight at 4°C followed by the incubation with Alexa Fluor-488-conjucated anti-rabbit secondary antibody, Fluor-568-conjugated anti-mouse secondary antibody (Molecular Probes) for 3 h at room temperature. The nuclei were counterstained in blue with 4’, 6-diamidino-2-phenylindole (DAPI; 5 μg ml^−1^, Molecular Probes) and mounted in Vectashield (VECTOR LABORATORIES). The images were visualized with an inverted MRc5 Carl Zeiss fluorescence microscope (Thornwood, NY) and OLYMPUS CLASM FV-100.

### Adenovirus-mediated overexpression of PDK4 in VSMCs

Recombinant adenovirus expressing PDK4 was provided by Dr. Young-Bum Kim (Harvard Medical School, MA). For details of Adenovirus-mediated overexpression of PDK4 in VSMCs, refer to the supplementary material method.

### GST pull-down assay

Full-length human *SMAD1*, mouse *Smad5* and rat *Smad8* were inserted into the EcoR1 and Xho1 sites of the pGEX4T-1 vector and amplified in *E. coli* BL21. Proteins were quantified using Coomassie Blue staining. [^35^S]-Methionine labeled-mouse PDK4, human SMAD1, mouse SMAD5, rat SMAD8 proteins were synthesized *in vitro* using TnT Quick Coupled Transcription/Translation System (Promega). GST and GST fused proteins were incubated with Glutathione-Sepharose TM4B (GE Healthcare) at room temperature for 20 min. Sepharose beads were washed with PBS and incubated with [^35^S]-methionine-labeled proteins in binding buffer (25 mM HEPES, pH 7.6, 1 mM DTT, 100 mM NaCl, 0.2 mM EDTA, 1.5% BSA, 20% glycerol) at 4 °C for 12 h. Samples were separated on NuPAGE gel (Invitrogen) and detected.

### Co-immunoprecipitation

Cells were harvested at 1000 rpm for 10 min, washed with PBS, and lysed with CoIPLysis buffer (1% Triton X-100 containing phosphatase and protease inhibitors). The cell extracts were incubated with PBS-washed ANTI-FLAG® M2-Agarose beads at 4°C overnight. After washing with lysis buffer and PBS, SDS-loading buffer with β-mercaptoethanol was added and the samples were boiled for 5 min. Proteins were separated by SDS-PAGE and detected by ODYSSEY (LI-COR).

### *In vitro* kinase assay

The GST-SMAD fusion proteins were used as substrates. GST-SMD fragments were incubated with recombinant PDK4 in kinase buffer and 20 μM ATP at 37 °C for 30 min. Kinase reactions were terminated by addition of NuPAGE loading buffer, heated at 70 °C for 10 min, and separated by NuPAGE. Dried gels were exposed to film using an Image Reader FLA-3000 series (FUJUFILM).

### Oxygen consumption rate measure

C2C12 cells were plated at 2.0 × 10^4^ cells / well in XF-24 cell culture microplate (Seahorse Bioscience, North Billerica, MA, USA). Oxygen consumption rate (OCR) was measured the next day using a XF-24 Analyzer (Seahorse Bioscience). Cells were equilibrated for 1 hour at 37 °C in XF Assay Medium (Seahorse Bioscience) supplemented with 25mM glucose and 1 mM sodium pyruvate (pH 7.4) before any measurement. Oligomycin (1 μM), CCCP (5 μM) and Rotenone (1 μM) mixture agents were injected to each well consecutively.

### Mitochondrial ROS assay^53^

Human VSMCs were allowed to attach on glass cover slips in 6-well plate at a density of 2 × 10^5^ cells per well. Cells were infected with adenovirus expressing PDK4 or CMV for 3 h and incubated with growth medium for 48 h. And the other cells were grown to confluence about 95% after then further incubated in the absence or presence of Pi for the indicated times. Both of cells were cultured with MitoTracker Red–CM–H_2_XROS (M7513, Invitrogen) at a 50 nM final concentration followed by incubation for 30 min in a CO_2_ incubator. Replace the normal media and detected by fluorescence microscope for 5s expose.

### Micro-CT analysis

To determine the three-dimensional bone structure *in vivo*, histomorphometric analyses were performed using a Micro-CT system (eXplore Locus SP scanner GE Healthcare) at 8 μm resolution. For details of micro-CT analysis, refer to the supplementary material method.

### Statistical analysis

Data are expressed as means ± S.E.M. Statistical analyses were performed using an unpaired Student’s *t*-test. A value of *P* < 0.05 was considered statistically significant.

## Additional Information

**How to cite this article**: Sun Joo, L. *et al.* Pyruvate Dehydrogenase Kinase 4 Promotes Vascular Calcification via SMAD1/5/8 Phosphorylation. *Sci. Rep.* **5**, 16577; doi: 10.1038/srep16577 (2015).

## Supplementary Material

Supplementary Information
