# Corrigendum: Pyruvate Dehydrogenase Kinase 4 Promotes Vascular Calcification via SMAD1/5/8 Phosphorylation

**DOI:** 10.1038/srep18552

**Published:** 2016-01-27

**Authors:** Sun Joo Lee, Ji Yun Jeong, Chang Joo Oh, Sungmi Park, Joon-Young Kim, Han-Jong Kim, Nam Doo Kim, Young-Keun Choi, Ji-Yeon Do, Younghoon Go, Chae-Myeong Ha, Je-Yong Choi, Seung Huh, Nam Ho Jeoung, Ki-Up Lee, Hueng-Sik Choi, Yu Wang, Keun-Gyu Park, Robert A. Harris, In-Kyu Lee

Scientific Reports 5: Article number: 16577; 10.1038/srep16577published online 11122015; updated: 01272016

The original version of this Article contained a typographical error in the spelling of the author Chae-Myeong Ha which was incorrectly given as Chae-Myung Ha. This has now been corrected in the PDF and HTML versions of the Article.

