# Supplementary material for: Pyruvate Dehydrogenase Kinase 4 Promotes Vascular Calcification via SMAD1/5/8 Phosphorylation
Source: Sci Rep. 2015 Nov 12;5:16577. doi: 10.1038/srep16577 (PMC4642318; doi:10.1038/srep16577)
Supplement: Supplementary Information [file srep16577-s1.pdf]

Supplementary Information

Pyruvate Dehydrogenase Kinase 4 Promotes Vascular Calcification via SMAD1/5/8 Phosphorylation

Sun Joo Lee†, Ji Yun Jeong†, Chang Joo Oh†, Sungmi Park, Joon-Young Kim, Han-Jong Kim, Nam Doo Kim, Young-Keun Choi, Ji-Yeon Do, Younghoon Go,Chae-Myung Ha, Je-Yong Choi, Seung Huh, Nam Ho Jeoung, Ki-Up Lee, Hueng-Sik Choi, Yu Wang, Keun-Gyu Park, Robert A. Harris* and In-Kyu Lee*

†These authors contributed equally to this work.

Corresponding authors

Prof. In-Kyu Lee
Division of Endocrinology and Metabolism,
Kyungpook National University School of Medicine, Daegu, Republic of Korea, 700-721
Tel: +82-53-420-5564
Fax: +82-53-426-2046
E-mail: leei@knu.ac.kr

Prof. Robert A. Harris
Department of Biochemistry and Molecular Biology, Indiana University School of Medicine and Roudebush VA Medical Center
1481 W. 10th Street, Indianapolis, IN 46202-5122, USA
Tel: +1-317-988-4544
Fax: +1-317-988-3180
E-mail: raharris@iu.edu

Supplementary Table

Supplementary Table S1. Medical information for patients who had been evaluated for vascular calcification based on the immunohistochemical staining of artery

Patients No.	Age/ Sex	Diagnosis	Operation	Diabetes	Hyper tensio n	Renal failure
1	68/F	Aortoiliac occlusive disease	Both EIA stents CFA Patch angioplasty	Yes	Yes	No
2	66/M	Aortoiliac occlusive disease	Both EIA stents CFA Patch angioplasty	No	No	Yes
3	72/M	Rt. carotid artery stenosis	CEA and patch angioplasty	No	Yes	No
4	73/M	Rt. carotid artery stenosis	Patch angioplasty	No	No	No
5	57/M	Lt. femoral artery stenosis	CFA endarterectomy and patch angioplasty	Yes	Yes	Yes
6	60/F	Rt. carotid artery stenosis	CEA and patch angioplasty	Yes	Yes	No
7	73/M	Rt. carotid artery stenosis	CEA and patch angioplasty	No	Yes	No
8	64/M	Rt. carotid artery stenosis	CEA and patch angioplasty	No	Yes	No

EIA, external iliac artery; CFA, common femoral artery; CEA, carotid endarterectomy

Supplementary Figures

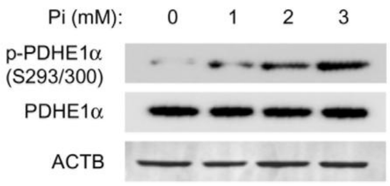

Supplementary Figure S1. The phosphorylation of PDHE1α increased during calcification. Western blot of total PDHE1α and p-PDHE1α during Pi-induced calcification in human VSMCs (n=4).

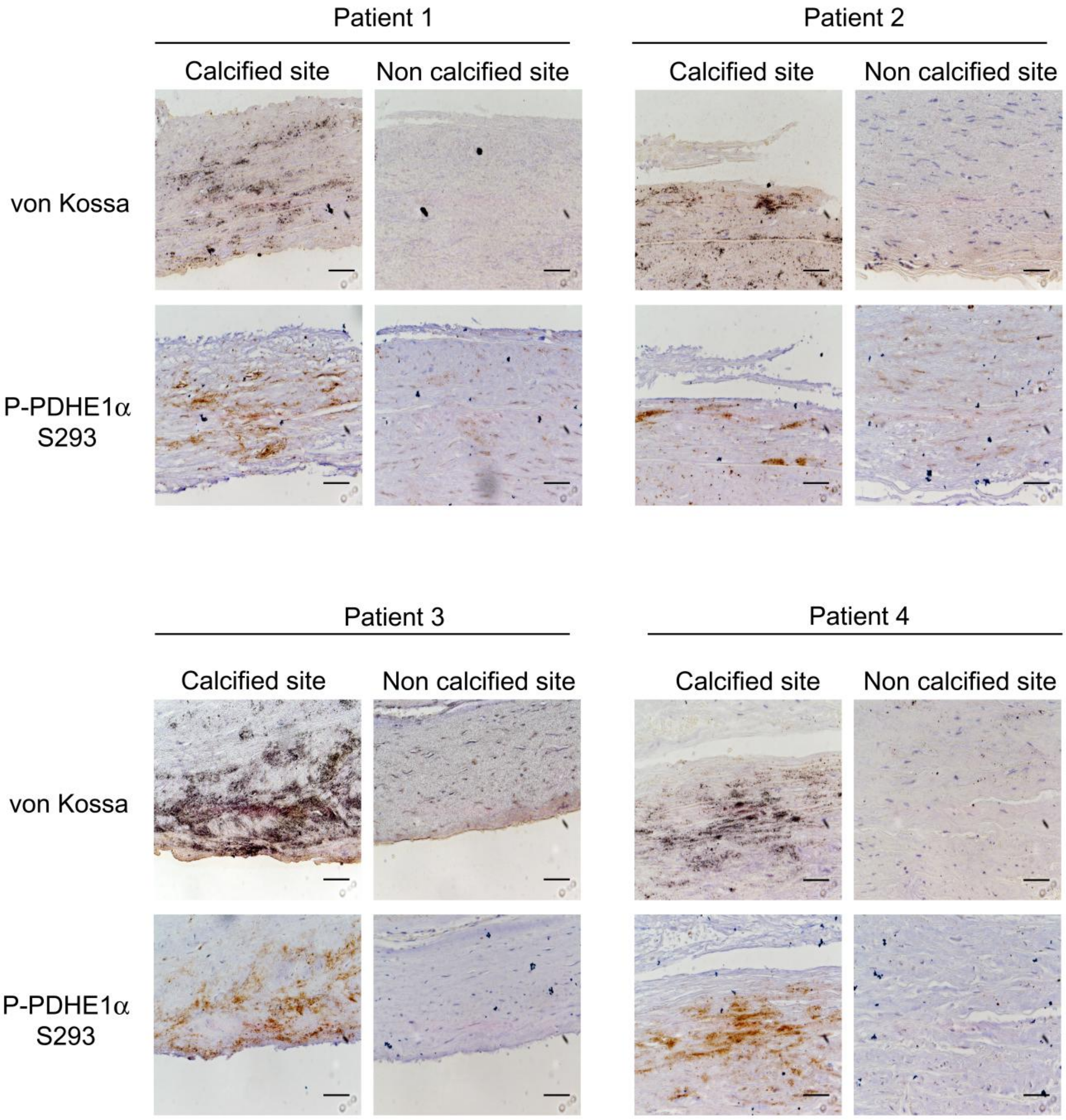

Supplementary Figure S2. The pattern of PDK coincides in the calcified area. Immunohistochemistry of p-PDHE1α S293 in a calcified site compared to a non-calcified site from each patients, respectively.

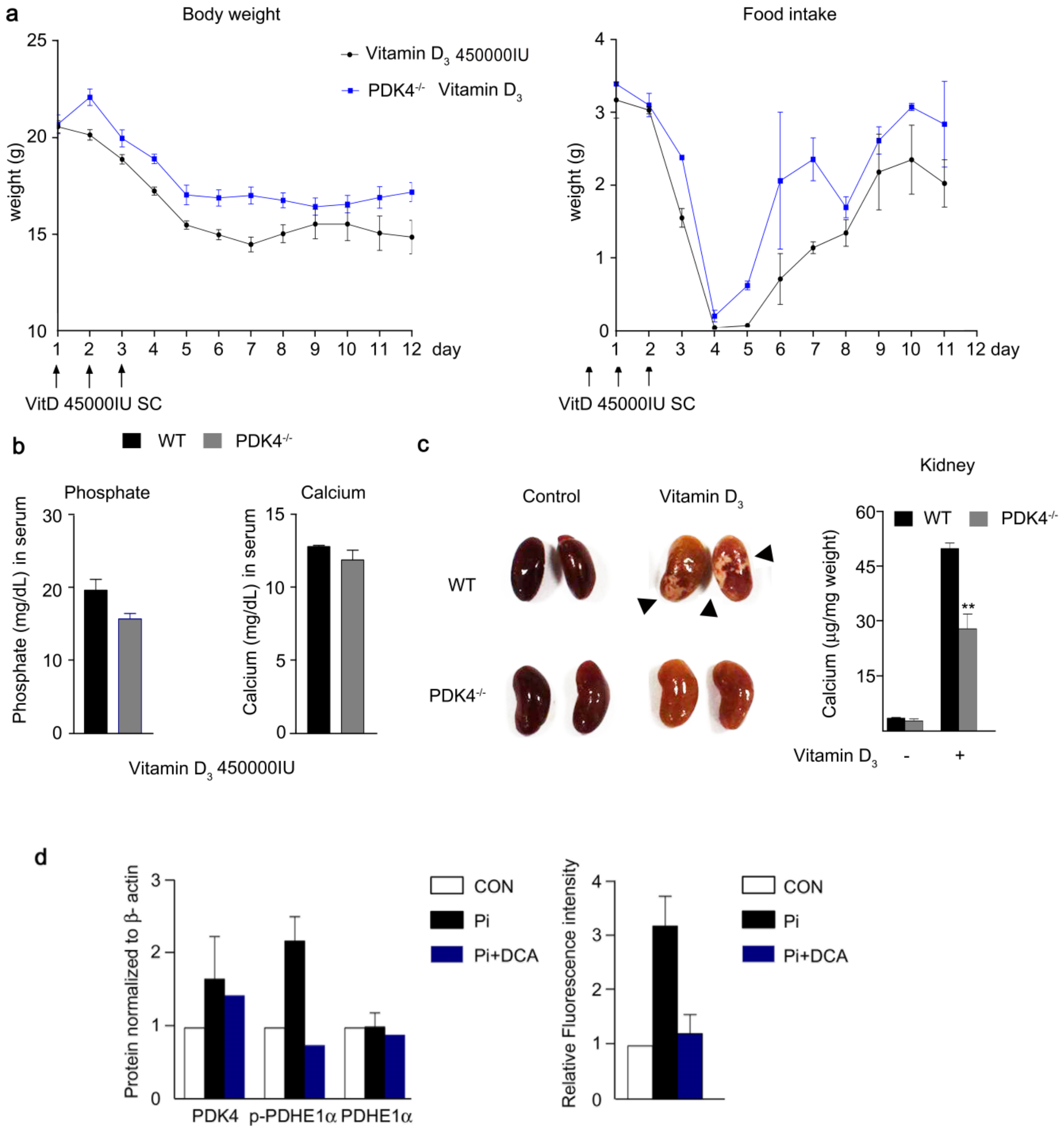
a
Body weight
Vitamin D3 450000IU
PDK4-/- Vitamin D3
weight (g)
day
VitD 45000IU SC
Food intake
weight (g)
day
VitD 45000IU SC
b
WT
PDK4-/-
Phosphate
Phosphate (mg/dL) in serum
Calcium
Calcium (mg/dL) in serum
Vitamin D3 450000IU
c
Control
Vitamin D3
WT
PDK4-/-
Kidney
Calcium (μg/mg weight)
WT
PDK4-/-
Vitamin D3
d
Protein normalized to β- actin
PDK4
p-PDHE1α
PDHE1α
CON
Pi
Pi+DCA
Relative Fluorescence intensity
CON
Pi
Pi+DCA

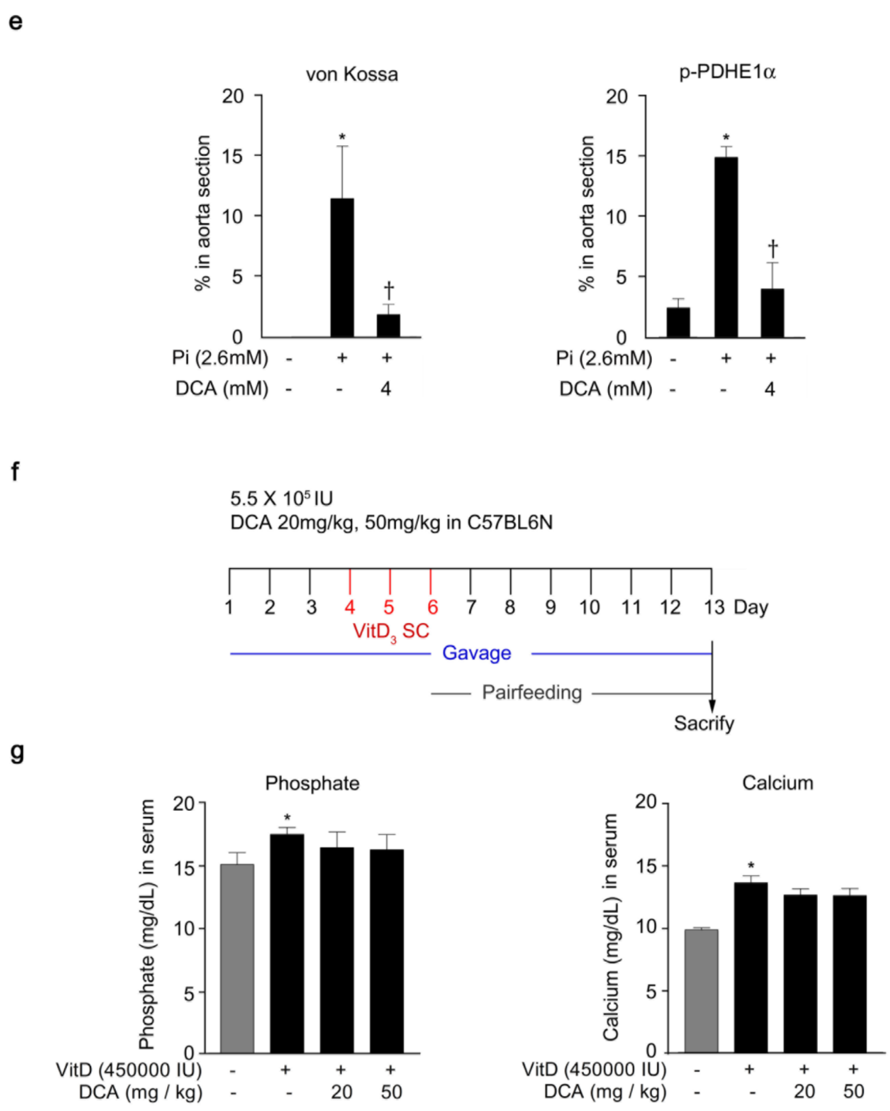

Supplementary Figure S3. Physiological parameters in mouse model of Vitamin D_3-induced aortic calcification. **(a)** Body weight and food intake. **(b)** Phosphate and calcium in serum. **(c)** Kidney morphology (left) and calcium score (right). **(d)** quantification of Western blot analysis (left) and immunofluorescence analysis (right, green) for p-PDHE1α S293/S300 in human VSMCs on day 3 after Pi treatment with or without DCA **(e)** quantification of von Kossa staining (left) and p-PDHE1α s293 (right) of *ex vivo* ring culture under the Pi conditions (n=3 rings per group). **P*<0.05 untreated stained section, †*P*<0.05 compared with Pi-treated stained section. **(f)** Overview of the experiment design for the animal study. Calcification was induced by subcutaneously injection of vitamin D_3 (cholecalciferol) for 3 days and DCA was given in drinking water administered to mice by gavage for 13 days as indicated. **(g)** Effect of DCA on phosphate level in VitD_3 model. (n=5 per group, n=3 per control group)**P*<0.05 compared with control.

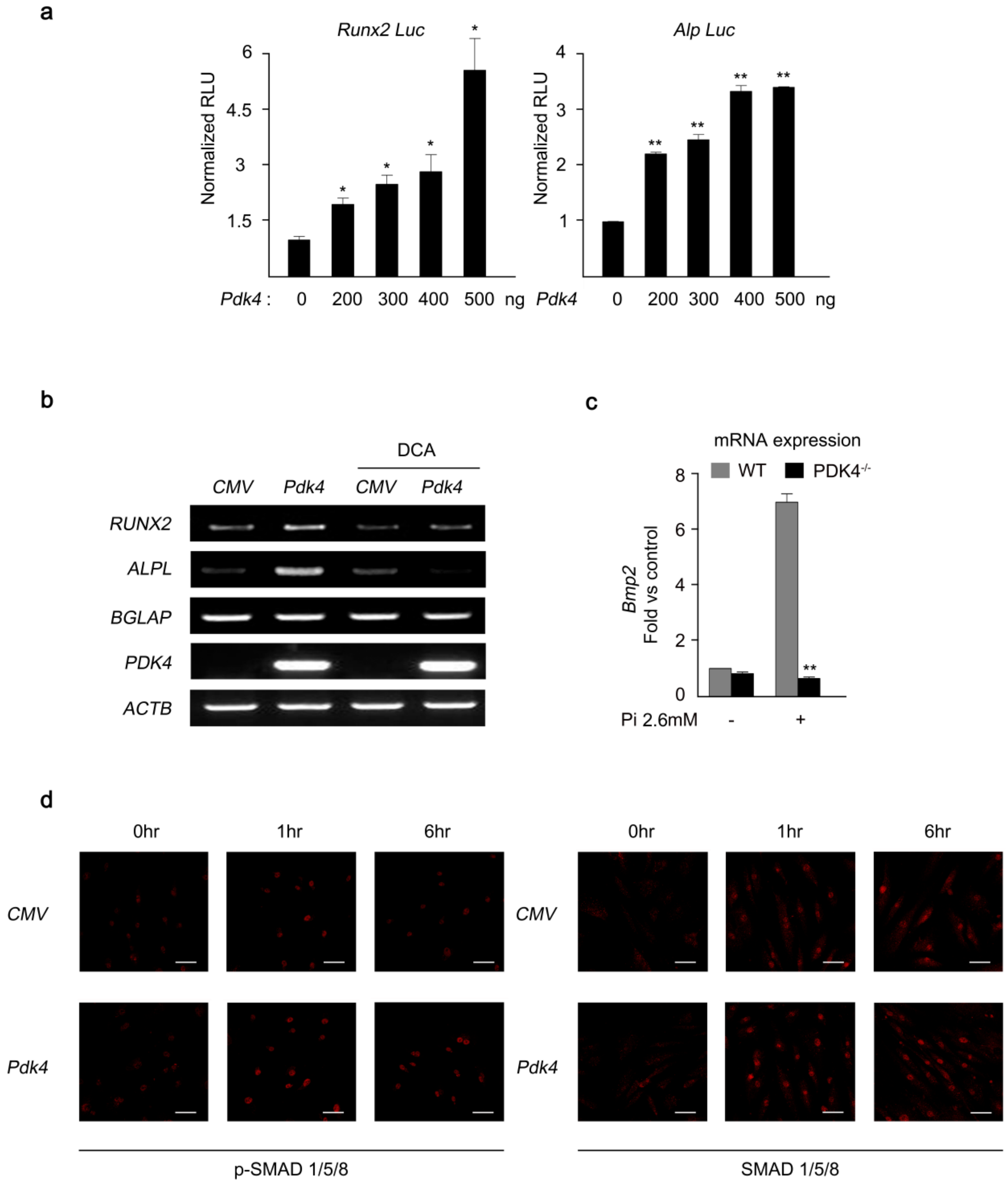

Supplementary Figure S4. PDK4 enhances SMAD signaling. (a) Luciferase activity measured in HEK293 cells after transfection with a *Pdk4* vector and luciferase reporter constructs for fragments of *Runx2* (-4615/+60), *Alpl* (1.9 kb) (n=3). **P*<0.05 compared with control. **(b)** RT-PCR analysis of osteogenic gene expression in human VSMCs infected with Ad-*CMV* or Ad-*Pdk4* with DCA. **(c)** RT-PCR analysis of *Bmp2* gene expression in VSMCs from WT and PDK4$^{-/-}$ mice cultured with or without Pi (n=3). ***P*<0.01 compared with WT. **(d)** Immuno-staining for p-SMAD1/5/8 and SMAD1/5/8 in human VSMCs cultured with BMP2 treatment after infection with Ad-*CMV* or Ad-*Pdk4*. Scale bar=50µm

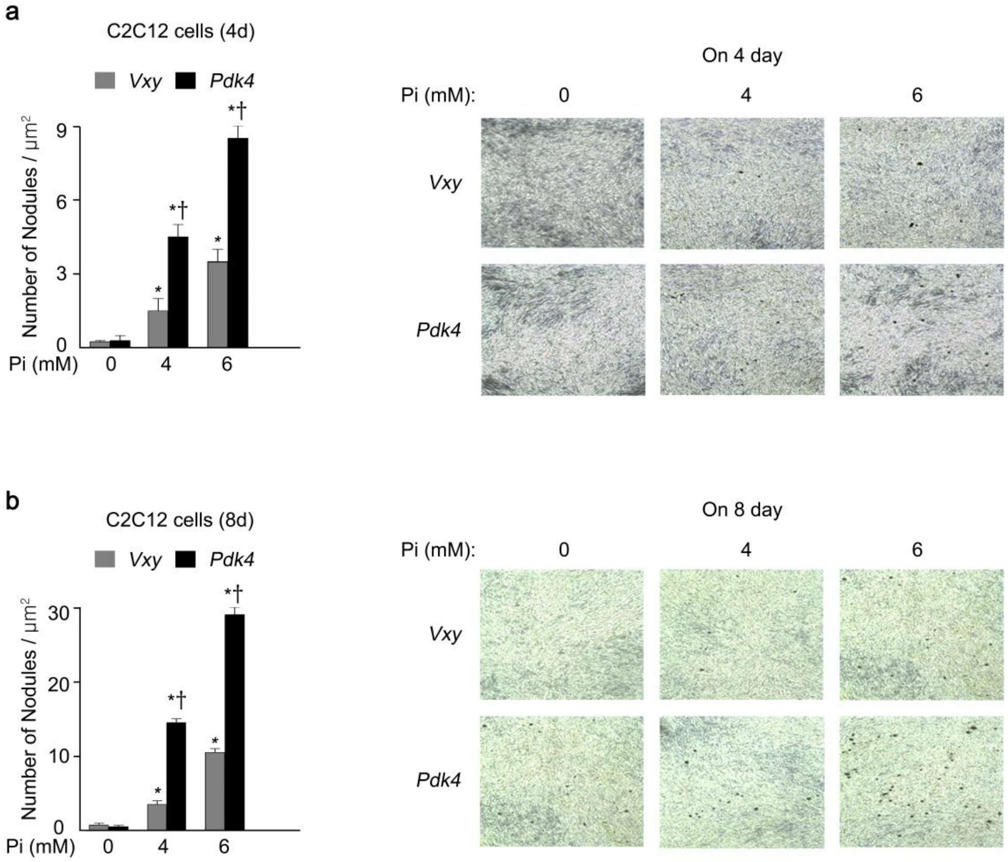

Supplementary Figure S5. Calcifications were enhanced by PDK4 overexpression in C2C12 cells. (a-b) Quantification of the number of nodules (left) and the representative images (right) on day 4 (a) and day 8 (b), respectively (n=3). **P*<0.05 compared with untreated control. †*P*<0.05 compared with *Vxy*.

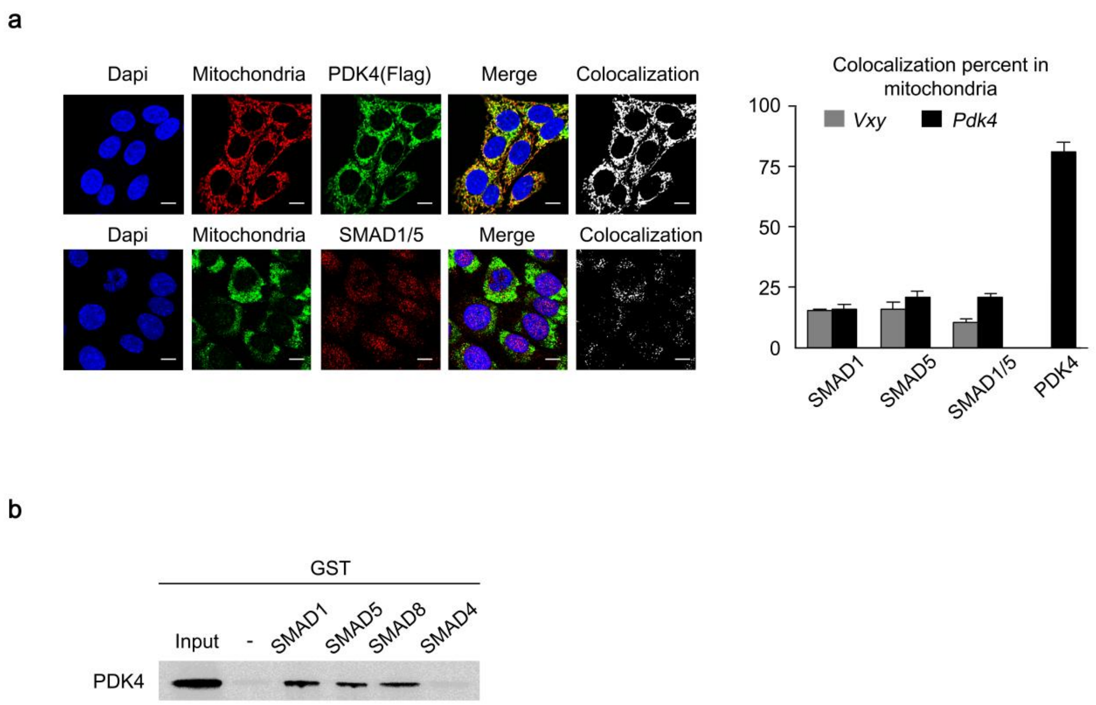

Supplementary Figure S6. PDK4 directly interacts with SMAD1/5/8. (a) Immunofluorescence images (left) and its quantification (right) of PDK4 (green, upper panel) and mitochondrial SMAD1, SMAD5, SMAD1/5 (red, lower panel) in C2C12 cells infected with control retrovirus or *Pdk4* expressing retrovirus. Nuclei and mitochondrial were stained by DAPI (blue) and Cox IV (red in upper panel and green in lower panel), respectively (n=5). Scale bar=20μm **(b)** GST-pull down assays with GST-SMADs and ^{35}S-labelled PDK4.

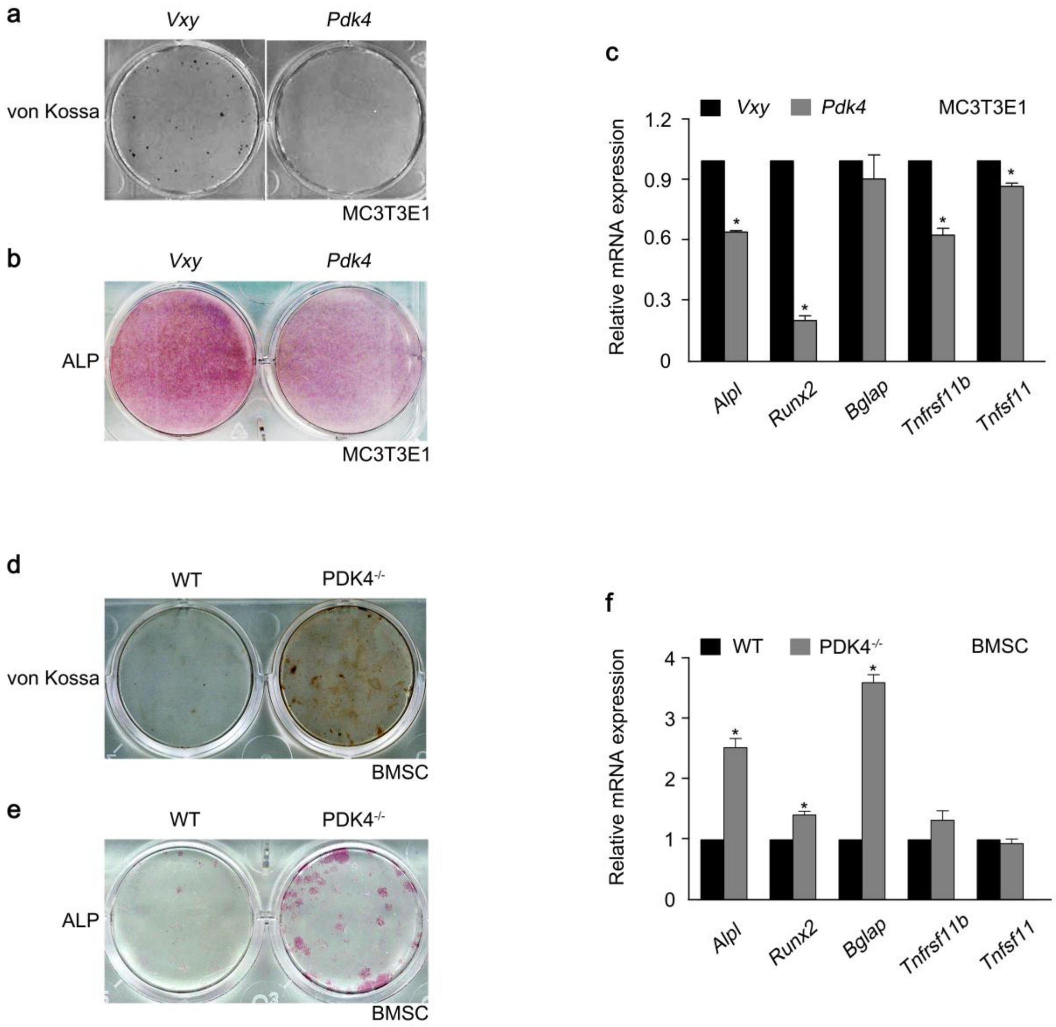

Supplementary Figure S7. PDK4 knockdown does not adversely affect normal bone remodeling. (a-c) Differentiation of MC3T3E1 cells infected with control retrovirus (*Vxy*) and *Pdk4* expressing retrovirus. (a) von Kossa staining after 36days in culture. (b) ALP staining on 8 day. (c) mRNA analysis of *Alpl*, *Runx2*, *Bglap*, *Tnfrsf11b*, and *Tnfsf11*(also known as *RANKL*) by RT-PCR on day 6. **(d-f)** Differentiation of BMSCs isolated from WT and PDK4$^{-/-}$ mice. (**d**) von Kossa staining on 14 days in culture. (e) ALP staining on 3 day. (f) mRNA analysis of *Alpl*, *Runx2*, *Bglap*, *Tnfrsf11b*, and *Tnfsf11* in these cells on 6 days. *P<0.05 compared with *Vxy* or WT

Supplementary Methods

Bone marrow stromal cell (BMSC) culture

The bone marrow stromal cells were obtained from C57BL/6J female mice. After the removal of red blood cell and filtering through 70 μm nylon mash (BD Falcon, 352350), the cells were cultured in 10% FBS α-MEM for 3 days. BMSCs were cultured in α-MEM with 10% FBS including β-glycerol phosphate (8 mM) and ascorbic acid (50 μg ml^{-1}). After 3 days, the cells were fixed with 4% PFA and stained with ALP staining solution (SIGMA, No. 86). On 4 day, the cells were fixed with 4% PFA and stained with 5% silver nitrate solution (von Kossa staining).

Cell culture and transient transfection.

Mouse VSMCs were isolated from the thoracic aortas of 5 week old male C57BL/6J mice. Cells were used for experiments after 4 to 5 passages. Human VSMCs were isolated from the thoracic aortas of kidney transplantation donors. The tissue on the inner side of the aorta was removed and the cells were used for experiments after 5 to 7 passages. The tissue was trimmed and washed in DMEM (high glucose (HG, 4500 mg/L) without PhenolRed, Hyclone), transferred to DMEM-HG media supplemented with 20% FBS, and cut into 1 mm cubes. The aorta was incubated in enzyme digestion solution (5 mL per 10 aortas) containing elastase (Sigma, E7885), collagenase type 1 (Sigma, C0130), trypsin inhibitor from glycine max (Sigma, T6414), bovine serum (BSA) for 20 min. Pellets obtained by letting the cells sediment were suspended in DMEM-HG media with 20% FBS.

HEK293 cells were obtained from the American Type Culture Collection (ATCC). Cells were maintained with DMEM (Gibco BRL), supplemented with 10% fetal bovine serum (FBS; Gibco BRL). C2C12 cells were obtained from the ATCC. Cells were maintained with DMEM-HG (supplemented with 10% FBS). For transient transfection, HEK293 cells (4-8 x 10^4 cells per well) were seeded in 24-well plates, grown overnight, and then transiently transfected with the reporter plasmids (200 ng per well) together with or without expression plasmids (100-200 ng per well each one). pcDNA3.0 plasmid was used for the normalization of the total amount of transfected DNA (500 ng of total plasmid DNA per well). Transfection was performed using Mirus reagent (Mirus Bio LLC) following the manufacturer's instructions. CMV-galactosidase plasmid (150 ng per well) was used as an internal control to normalize transfection efficiency.

Quantification of Calcium Deposition

For measuring calcium deposited in the extracellular matrix of the cells, the cells were washed with PBS and then decalcified with 0.6N HCl overnight and calcium contents in the supernatant were determined colorimetrically by the *o*-cresolphthaleincomplexone method (Bioassay System,

Cat.DICA500). The remaining cells were washed with PBS and solubilized with lysis buffer (0.1N NaOH and 0.1% SDS) and total protein contents were determined by the bicinchoninic acid (BCA) method (Pierce). Total calcium content was normalized by total protein content for each culture. For calcium quantification in aortic tissues, aortic segments were weighed and decalcified with 0.6N HCl for 24 h, and then calcium content was determined according to the above procedure and the data were expressed as calcium per mg of dry aortic tissues

Adenovirus-mediated overexpression of PDK4 in VSMCs

Recombinant adenovirus expressing PDK4 was provided by Dr. Young-Bum Kim (Harvard Medical School, MA). The recombinant adenovirus was amplified in HEK-293 cells and purified using CsCl (Sigma) gradient centrifugation. The preparation were collected and desalted, and the titers were determined using Adeno-X Rapid titer according to the manufacturer's instructions (BD Bioscience). VSMCs were infected with adenoviruses expressing PDK4 or GFP protein (control) at various multiplicities of infection (MOI) for 3 h and incubated with growth medium supplemented with or without various concentrations of Pi and DCA. The infection efficiency was determined by fluorescence intensity of GFP.

Binding model prediction of PDK4 and SMAD5

The structure of PDK4 in complex with ADP was obtained from the Protein Data Bank (http://www.pdb.org, pdb code: 2E0A). Discovery Studio3.5 (http://www.accelrys.com) with MODELLER was used for the homology model building of SMAD5. The template structure of SMAD1 (pdb code: 1KHU) was used to build the homology model. The initial PDK4 and SMAD5 structures were minimized by the steepest descent method for 5,000 iterations using the CHARMm force field (Accelrys, San Diego, CA, USA). To predict PDK4-SMAD5 binding model, the ZDOCK and RDOCK programs in Discovery Studio 3.5 (Accelrys) were used. ZDOCK for PDK4-SMAD5 docking included using a filter feature to specify the residues involved in the binding interface since the autophosphorylation site of PDK4 and the SMAD5 phosphorylation site are expected to be involved. The top 2,000 predictions for each case were generated using ZDOCK at a 6° rotational sampling density with each of the scoring functions. Clusters of similar docking pose and the top-scoring cluster were selected for the PDK4-SMAD5 complex. The molecular graphics for the binding-model structure were generated using the PyMol package (http://www.pymol.org).

Micro-CT analysis

To determine the three-dimensional bone structure *in vivo*, histomorphometric analyses were performed using a Micro-CT system (eXplore Locus SP scanner GE Healthcare) at 8 μm resolution.

The morphometric parameters were determined using eXplore MicroView program (GE Healthcare; version 2.2). For the femur analysis, the scanned regions were confined to the distal metaphysis, extending proximally 1.7 mm from the proximal tip of the primary spongiosa.

Immunohistochemistry

The vessels were routinely processed for paraffin embedding at a thickness of 10 μm after 4% paraformaldehyde fixation for the analysis. The sections were de-paraffinized and antigens were retrieved using an IHC-Tek Epitope Retrieval Steamer Set (IHC World (IW-1102), Woodstock, MD, USA) in 0.01 M citrate buffer (pH 6.0) at 95℃ for 40 min, followed by overnight incubation with antibodies against p-PDHE1 S293 and S300 (Calbiochem, Cat # AP1062, AP1064, AP1063, respectively) at 4℃ and detection using secondary antibodies with HRP, using the UltraVision LP Detection System (Thermo Scientific, Waltham, MA, USA). Nuclei were counterstained with Mayer's hematoxylin (Lillie's modification).
